# Supplementary material for: Purkinje cell axonal swellings enhance action potential fidelity and cerebellar function
Source: Nat Commun. 2021 Jul 5;12:4129. doi: 10.1038/s41467-021-24390-4 (PMC8257784; doi:10.1038/s41467-021-24390-4)
Supplement: Supplementary file 1 — Supplementary Information [file 41467_2021_24390_MOESM1_ESM.pdf]

Purkinje cell axonal swellings enhance action potential fidelity and cerebellar function.

Supplementary Information

Daneck Lang-Ouellette, Kim Gruver, Amy Smith-Dijak, François G. C. Blot, Chloe A. Stewart, Pauline de Vanssay de Blavous, Connie H. Li, Carter Van Eitrem, Charlotte Rosen, Phyllis L. Faust, Martijn Schonewille, and Alanna J. Watt

Supplementary Figs. 1-11

Supplementary Table 1-2

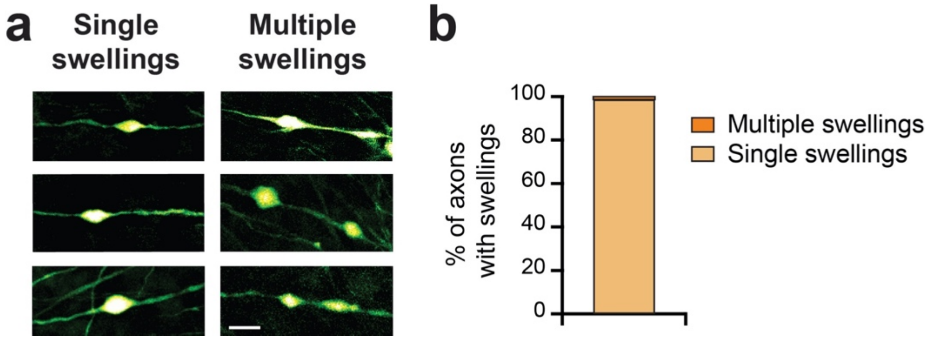

Supplementary Fig. 1. Majority of axons with swellings have single swellings.

(**a**) Sample images of axons with single swellings (left) and rare axons with multiple swellings (right). A total of 963 swellings were identified in 23 image acquisitions from N = 4 animals. Scale bar, 10 µm. (**b**) The majority of axons that have swellings have a single swelling in the granule cell layer (98.7%, or 950/963 of axons with swellings had single swellings). Axons with single axonal swellings were targeted for recording. Source data are provided as a Source Data file.

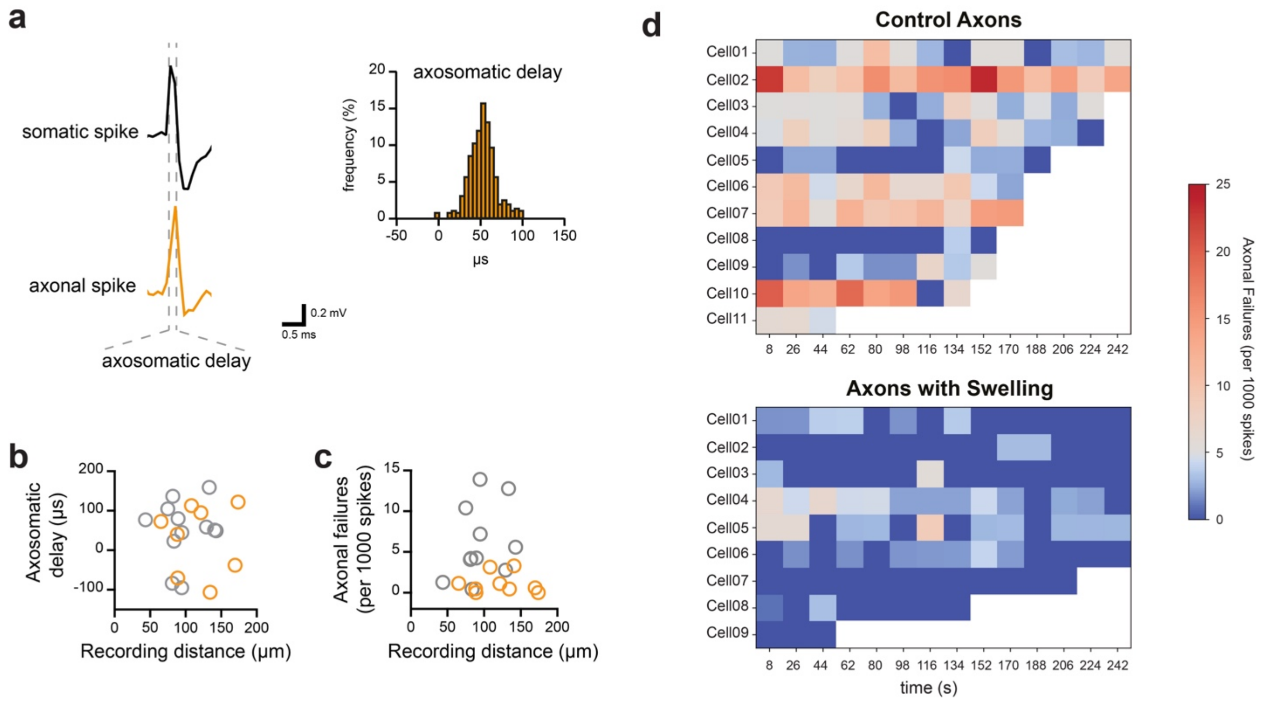

Supplementary Fig. 2. Propagation velocity is not altered in axons with swellings.

(**a**) Measurement of the axosomatic delay were taken by measuring the delay between the somatic and axonal spikes (example shown on left) over multiple spike pairings (right). For the recorded pair, 349 paired somatic and axonal action potentials were used to create the axosomatic distribution (right). (**b**) The axosomatic delay as a function of recording distance for control axons (grey circles) and for axons with swellings (orange circles). Axosomatic delay can be negative when the action potential reaches the axonal recording electrode before the somatic electrode, due to the spike initiation zone being located on the axon initial segment. There is no relationship between the axosomatic delay and the distance of the axonal recording from the soma for control axons or for axons with swellings, at least over the relatively short (< 200 μm) distances that we recorded from. (**c**) There is also no relationship between the axonal failure rates and the recording distance in control axons or axons with swellings. (**d**) Axonal failures as a function of time for each pair of cells recorded for control axons (top) and axons with swellings (bottom). Cells that display higher axonal failures tend to do so throughout their recording, and cells that display lower axonal failures tend to do so throughout their recording. Source data are provided as a Source Data file.

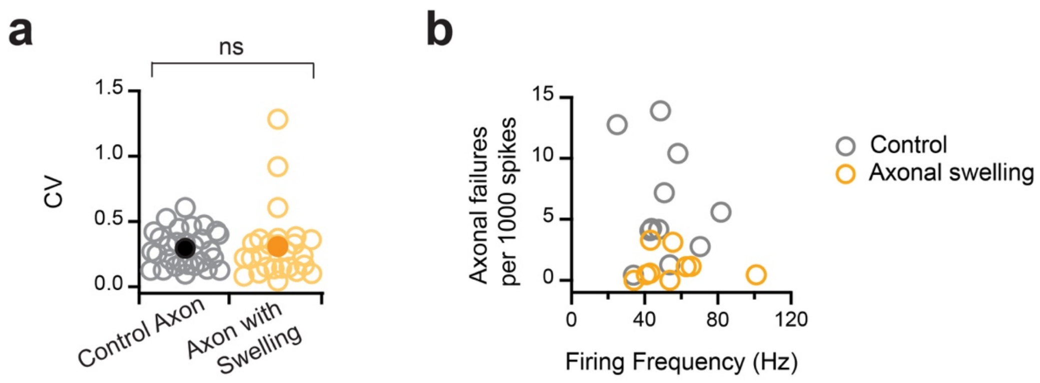

Supplementary Fig. 3. Axonal swellings and failure rate are not correlated with firing properties.

(**a**) The coefficient of variation (CV), a measure of the regularity of Purkinje cell firing is not different in neurons with and without axonal swellings. (CV: Control Axon: 0.293 ± 0.026; Axon with Swelling: 0.307 ± 0.053; not significantly different, P = 0.40). Control Axons, n = 29; Axon with Swelling, n = 26. (**b**) Axonal failures as a function of firing frequency. There is no significant correlation between firing rate and failure rate for either control axons or axonal swellings. Control axons, n = 11; Axons with swellings, n = 9. Data are presented as mean ± SEM (ns P > 0.05). Source data are provided as a Source Data file.

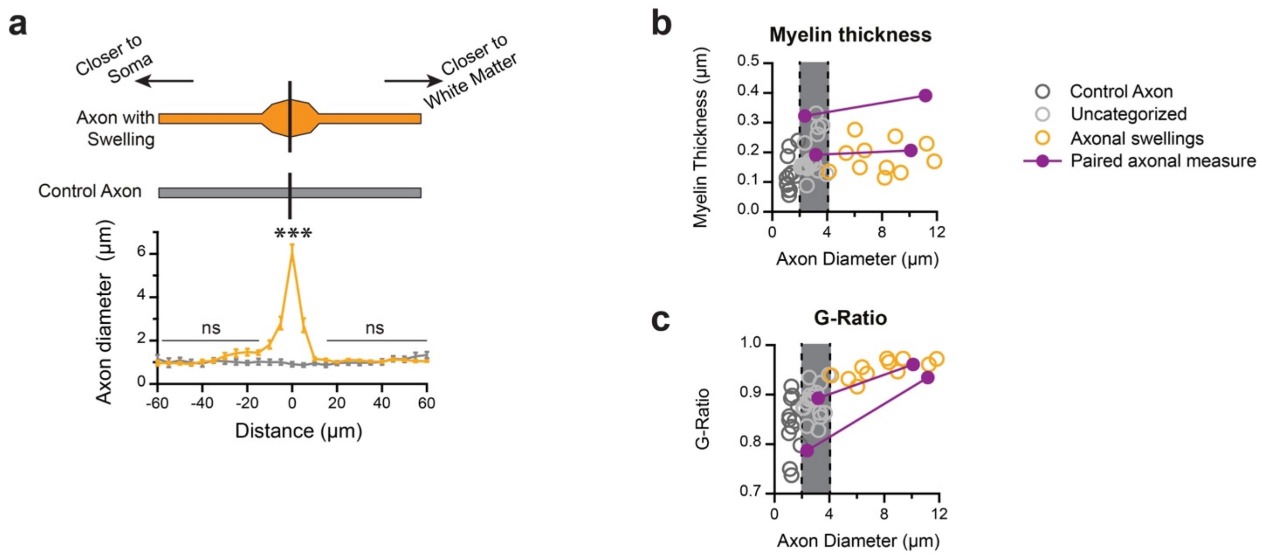

Supplementary Fig. 4. Axonal swellings occur on axons of similar diameters to control axons but have larger G ratios than control axons.

(**a**) To determine whether swellings occurred on axons that were already thicker, we measured the diameter of axons with swellings and those without from GFP-labeled axons using 2-photon microscopy, and found no significant difference in the diameter within 15 µm on either side of the centre of the axonal swelling (centred on the swelling; left: negative values are closer to the soma; right: positive values are closer to the white matter), and equivalent locations from control axons. The diameter of the axonal swelling was significantly larger than that of control axons (one-sided Mann-Whitney *U* test, P = 0.004; n = 15 axons with swellings, n = 13 control axons). (**b**) Myelin thickness was measured for axons (< 2 µm, dark grey), axonal swellings (> 4 µm, orange) and a mixed population of uncategorized axons and swellings (between 2 and 4 µm, light grey), which is plotted as a function of axon diameter. (**c**) G-ratio was measured for axons (< 2 µm, dark grey), a mixed population of axons and swellings, that are thus uncategorized (between 2 and 4 µm, light grey), and axonal swellings (> 4 µm, orange). Two instances where a swelling and an axon were connected in the image (e.g. **Fig. 3a**) are shown in purple, and follow the general observation that the g-ratio is larger for swellings than for axons. Data are presented as mean ± SEM (***P < 0.005, ns P > 0.05). Source data are provided as a Source Data file.

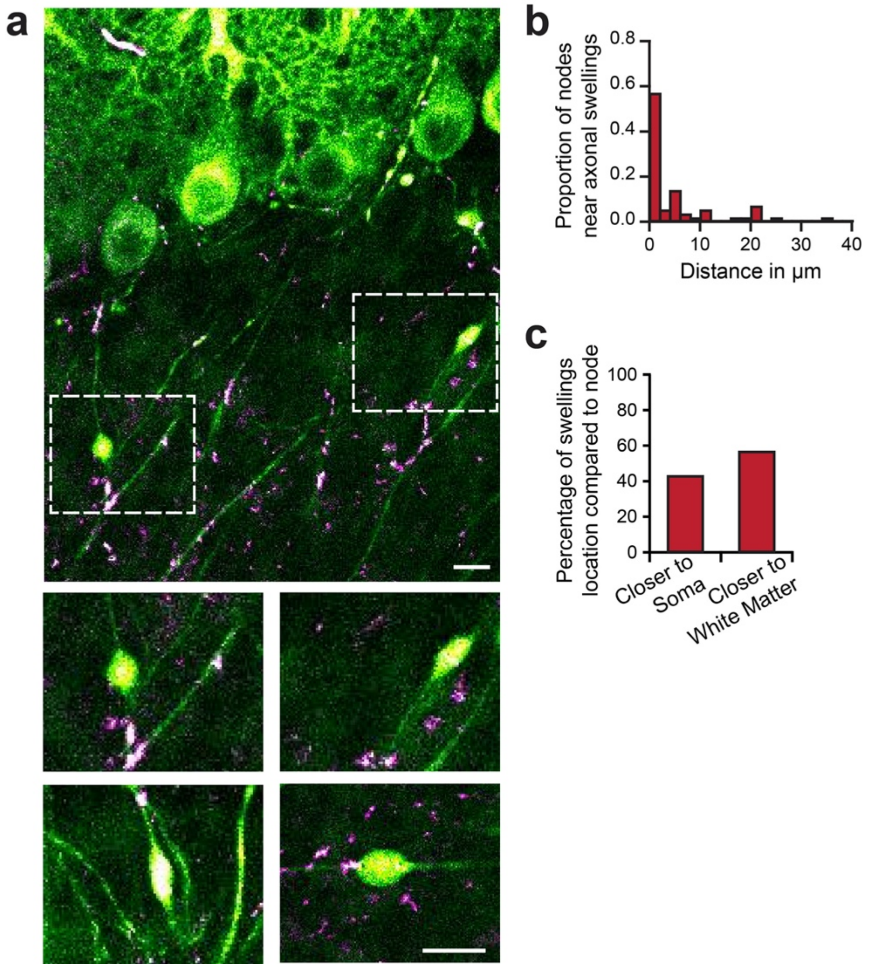

Supplementary Fig. 5. Axonal swellings are often located close to nodes of Ranvier.

(**a**) Examples of Purkinje cell axons (green) stained for the paranodal protein CASPR (purple). A total of 14 images were used for this data set. CASPR labeling was almost never observed directly on axonal swellings, suggesting that these structures are not typically located precisely at nodes of Ranvier. However, CASPR puncta were often observed near axonal swellings, suggesting that they were frequently close to nodes of Ranvier. Top: image shows Purkinje cells and several swellings. Second row: axonal swellings shown in top image (white boxes), expanded. Paranodal CASPR is near but no co-localized with swellings. Bottom row: further examples showing paranodal CASPR staining that is close (left) or very proximal (right) to axonal swellings. Scale bar, 20 µm (top); 10 µm (middle and bottom rows). Note that CASPR staining labels paranodal structures on non-Purkinje axons as well as Purkinje axons. (**b**) Nearly 50% of axonal swellings were located within 2 µm of CASPR puncta. (**c**) Axonal swellings did not show preferential up- or down-stream localization in comparison to CASPR puncta: roughly half were located closer to the soma and half closer to the white matter. N = 3 mice, n = 58 axonal swellings. Source data are provided as a Source Data file.

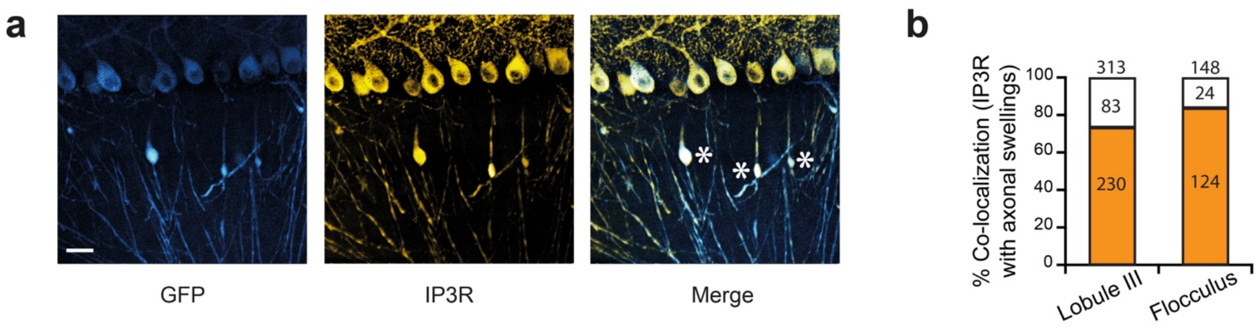

Supplementary Fig. 6. Axonal swellings express IP3Rs.

(**a**) Representative image showing the majority of axonal swellings (GFP, blue) colocalize with IP3R (yellow). Total image acquisition for Lobule III = 36 and flocculus = 16. Asterisks show axonal swellings co-labeled for IP3R in merged image (right). Scale bar, 20 μm. (**b**) Summary data showing that the majority of axonal swellings are positive for IP3R staining in both lobule III (left; n = 313 axonal swellings from N = 3 animals) and in the flocculus (right; n = 148 axonal swellings from N = 3 animals). Source data are provided as a Source Data file.

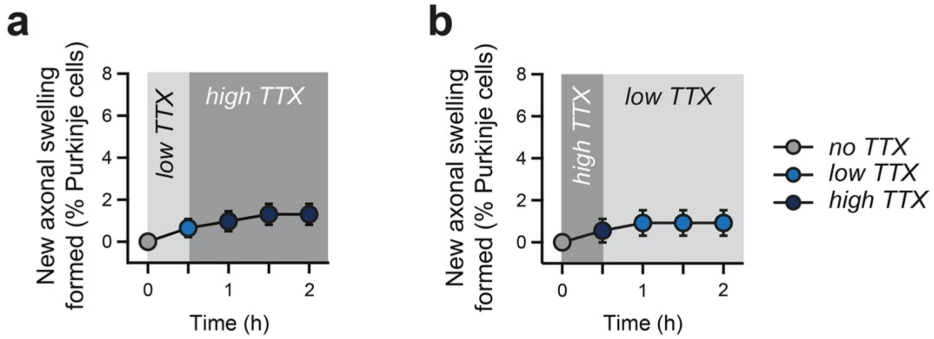

Supplementary Fig. 7. Insufficient exposure to low TTX will not lead to axonal swelling formation.

(**a**) To determine the minimum time course over which the formation of axonal swellings occurs, we perfused slices with *low TTX* for 30 minutes followed by 90 minutes of *high TTX* (that will block all action potentials). This duration was insufficient to induce the formation of axonal swellings (*low TTX followed by high TTX*: 1.30 ± 0.50 new axonal swellings in % of Purkinje cells; n = 8; not significantly different over time when compared to ACSF, repeated measure ANOVA, P = 0.74). (**b**) We then reversed the order of application, perfusing *high TTX* for 30 minutes which will completely block action potentials followed by 90 minutes of *low TTX* and found that no additional swellings were formed with this paradigm (*high TTX followed by low TTX:* 0.93 ± 0.06 new axonal swellings % of Purkinje cells; n = 6; repeated measure ANOVA, P = 0.65). The 90 minute duration of *low TTX* in the second paradigm likely produces relatively few failures because of the preceding *high TTX* compared to *low TTX* on its own for 2 hours (120 minutes). These data suggest that sufficient numbers of axonal failures are required for the formation of axonal swellings, and that this is not simply determined by the duration of the paradigm. Data are presented as mean ± SEM. Source data are provided as a Source Data file.

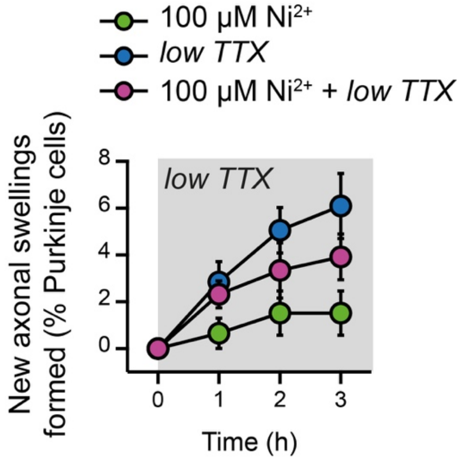

Supplementary Fig. 8. Partial blockade of new swelling formation with sub-saturating concentration of Ni^{2+}.

We used a sub-saturating concentration of $Ni^{2+}$to partially block voltage-gated calcium channels and found that it partially blocked the formation of swellings in the presence of *low TTX* (repeated measures ANOVA showed no effect of the conditions over time). Sub-saturating Ni^{2+} concentration partially blocks axonal swelling formation (100 mM Ni^{2+} + *low TTX*: 3.91 ± 0.98% new swellings; n = 6) compared to low TTX (*low* TTX: 6.09 ± 1.39%; n = 14) and Ni^{2+} only (100 mM Ni^{2+}: 1.51 ± 0.94%; n = 5; repeated measure ANOVA, P = 0.209). Data are presented as mean ± SEM. Source data are provided as a Source Data file.

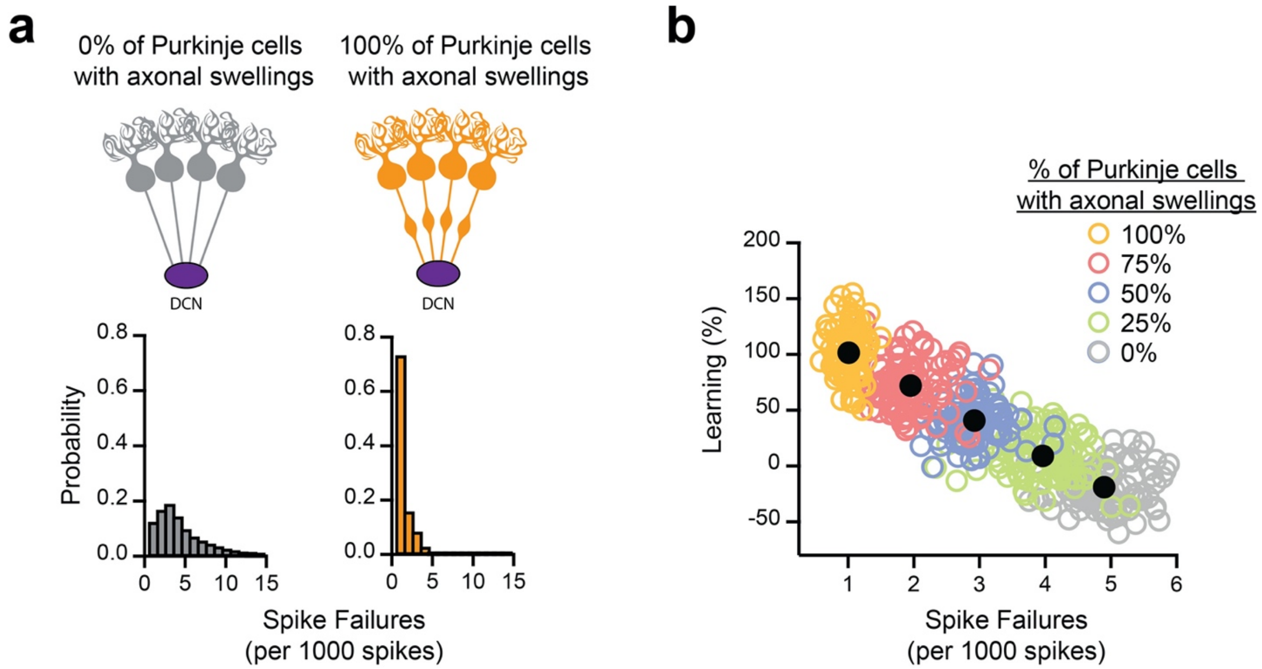

Supplementary Fig. 9. Monte Carlo simulation relating learning to axonal swellings in a network model.

(**a**) Percentage of spike failures for simulated output of a 200,000-Purkinje cell network (representing the whole cerebellum) that has either 0% of Purkinje cells with axonal swellings (left) or 100% (right). Each simulation was run 5000 times and the incidence of network output failures is shown in the histogram, using experimental data to populate the simulation. The probability of spike failures in a network is more likely to be closer to 0 with a greater percentage of axonal swellings. (**b**) Subsequent projection of the amount of learning on the Rotarod tasks given that the Monte Carlo simulation of the network of 200,000 Purkinje cells predicts behavior. Simulation was run sampling from experimentally-determined data 100 times (individual dots) for different percentages of Purkinje cells with axonal swellings (0, grey; 25, green; 50, blue; 75, rose; 100%, yellow). Note that the amount of learning observed varies with each run of the simulation but that overall the percentage of Purkinje cells with axonal swellings has a dramatic impact on learning. Source data are provided as a Source Data file.

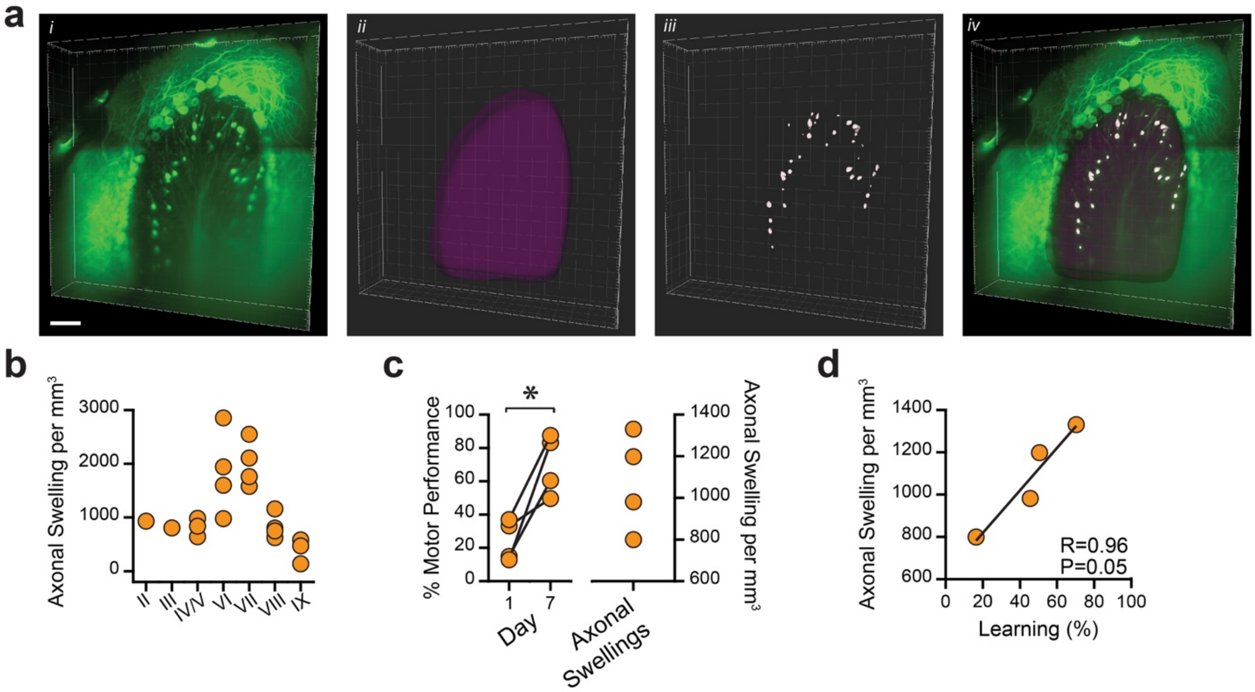

Supplementary Fig. 10. Light-sheet imaging reveals that the density of axonal swellings varies across lobule within the cerebellar vermis.

Left to right images: (**a**) Analysis pipeline. (*i*) Small vermal section from lobule II expressing GFP in Purkinje cells. (*ii*) Granule cell layer delineation, enabling axonal swellings to be detected in this area (area in purple). (*iii*) Automatic detection of axonal swellings in delineated area based on size, sphericity, and intensity. (*iv*) Merge of (*i- iii*) shows good detection of swellings. Scale bar, 100 µm. (**b**) The density of axonal swellings varies across vermal lobule. (**c**) Mice exhibit variable learning on the Rotarod task, and variable overall density of axonal swellings (two-sided paired Student's *T* test, P = 0.026). (**d**) The number of axonal swellings demonstrates a trend of varying proportionally to the overall axonal swelling density (including lobules where > 2 mice were imaged, two-sided Pearson's correlation, R = 0.96, P = 0.045). N = 4 mice. *P < 0.05 Source data are provided as a Source Data file.

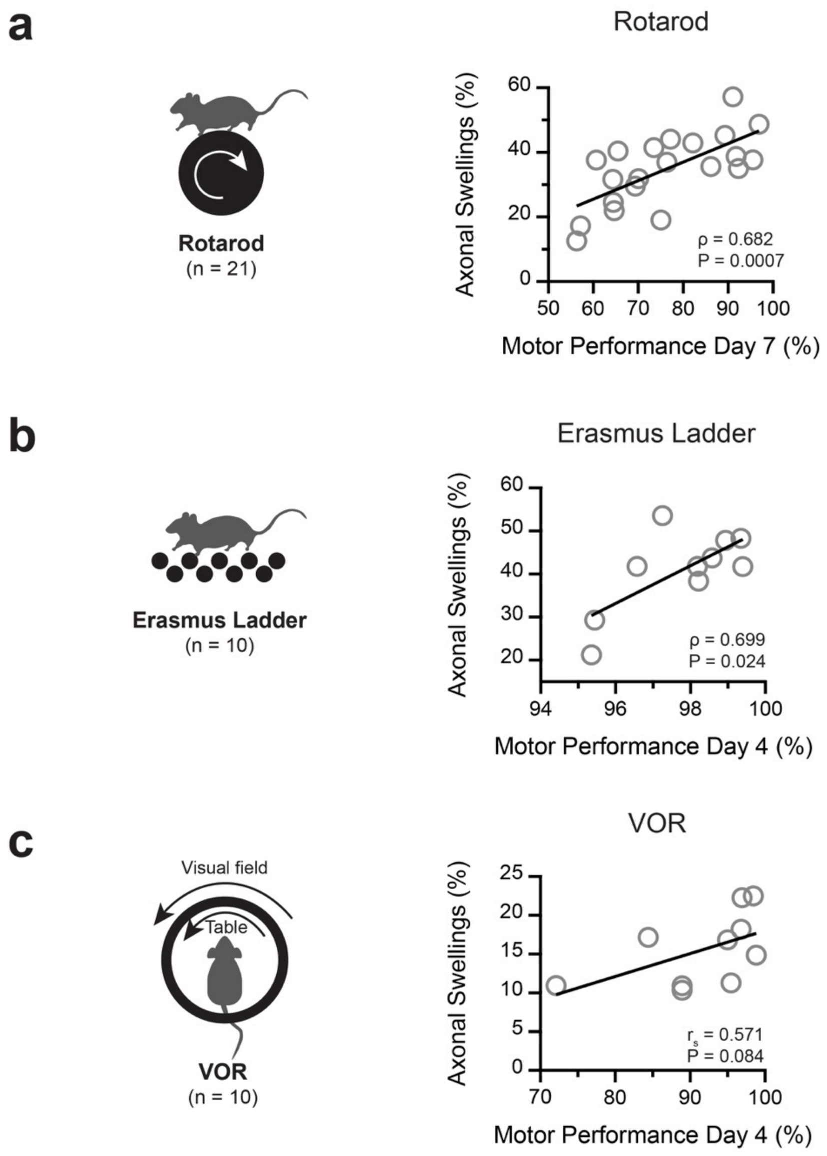

Supplementary Fig. 11. Axonal swelling density is also positively correlated with motor performance on the last day of assays. (**a**) Rotarod assay. The percentage of Purkinje cells with axonal swellings in anterior cerebellum is positively correlated with motor performance on Day 7. Each animal is represented with a single data point. Correlation measured using Pearson's Correlation (two-sided R = 0.682, P < 0.001). (**b**) Erasmus ladder. The percentage of Purkinje cells with axonal swellings in anterior cerebellum is positively correlated with motor performance on Day 4. Correlation measured using Pearson's Correlation (two sided R = 0.699, P = 0.024). (**c**) VOR phase reversal. The percentage of Purkinje cells with axonal swellings on their axons shows a weak correlation that is not significant with the VOR performance on the last day. Correlation measured using Spearman's Correlation (two-sided r_s = 0.571, P = 0.084).

Supplementary Table 1 Cellular and subcellular properties of axonal swellings and control axons.

Category	Control axons<br>Average ± SEM (range, n)	Axonal swellings<br>Average ± SEM (range, n)	P value
Location of Recordings (μm from soma)	95.3 ± 8.79 (43.5 – 142.8 μm; n = 11)	121.2 ± 12.43 (65.1 – 173.8 μm; n = 9)	0.098
Failure rates (per 1000 spikes) (Fig. 1c)	6.07 ± 1.36 (0.41 – 13.90; n = 11)	1.12 ± 0.41 (0 – 3.28; n = 9)	0.0023
Axonal Propagation Reliability at maximum frequency (% somatic spikes; Fig. 2c)	84.1 ± 4.85 (72.8 – 95.5; n = 5)	96.9 ± 0.63 (96.0 – 98.7; n = 4)	0.016
Maximum frequency (Hz; Fig. 2c)	128.0 ± 23.1	162.5 ± 6.3	0.44
Organelle density (% area)	27.04 ± 2.82 (2.53 – 55.14; n = 17)	32.34 ± 2.30 (20.02 – 55.47; n = 15)	0.162
g ratio	0.84 ± 0.16 (0.74 – 0.92; n = 12)	0.95 ± 0.005 (0.92 – 0.97; n = 13)	0.009
Firing rate (Hz)	45.16 ± 3.15 (23.88 – 82.75; n = 29)	44.32 ± 3.71 (16.75 – 106.16; n = 26)	0.22
CV	0.29 ± 0.03 (0.098 – 0.61; n = 29)	0.31 ± 0.05 (0.044 – 1.28; n = 26)	0.40

P values are reported for two-sided t tests when distributions are normal, or for Mann-Whitney *U* tests when distributions are not. The g ratio was measured only for a subset of axons where compact myelin could be measured reliably (due to fixation artefacts).

TTX Firing (Fig. 4c)	P-Value
**Within-Subject effect**	
Time*Condition	0.023
**Multivariate Tests (Wilk's Lambda)**	
Soma	<0.001
Axon	0.109
**Pairwise comparisons**	
Soma - Axon	
30 min	<0.001
45 min	<0.001
60 min	<0.001
75 min	<0.001
90 min	<0.001

TTX Concentration (Fig. 4e)	P-Value
**Within-Subject effect**	
Time*Condition	0.008
**Multivariate Tests (Wilk's Lambda)**	
No TTX	0.738
Low TTX	<0.001
High TTX	0.736
**Pairwise comparisons**	
Low TTX	
0h – 1h	<0.001
0h – 2h	<0.001
0h – 3h	<0.001
1h – 2h	<0.001
1h – 3h	<0.001
2h – 3h	0.009

Volume (Fig. 4g)	P-Value
**Within-Subject effect**	
Time*Condition	0.004
**Multivariate Tests (Wilk's Lambda)**	
Control Axons	0.471
Axons with Swelling	0.002
**Pairwise comparisons**	
Axon with Swelling	
0h – 1h	0.346
0h – 2h	0.007
0h – 3h	<0.001
1h – 2h	0.221
1h – 3h	0.003
2h – 3h	0.002

Nickel concentration (Fig. 5c)	P-Value
**Within-Subject effect**	
Time*Condition	0.011
**Multivariate Tests (Wilk's Lambda)**	
1mM Ni^{2+}	0.779
1mM Ni^{2+} + Low TTX	0.792
Low TTX	<0.001
**Pairwise comparisons**	
Low TTX	
0h – 1h	0.001
0h – 2h	<0.001
0h – 3h	<0.001
1h – 2h	0.005
1h – 3h	0.012
2h – 3h	0.443

Ca^{2+} Concentration (Fig. 5b)	P-Value
**Within-Subject effect**	
Time*Condition	<0.001
**Multivariate Tests (Wilk's Lambda)**	
0mM Ca^{2+}	0.245
0mM Ca^{2+} + Low TTX	0.162
2mM Ca^{2+} + Low TTX	<0.001
3mM Ca^{2+} + Low TTX	<0.001
**Pairwise comparisons**	
2mM Ca^{2+} + Low TTX	
0h – 1h	<0.001
0h – 2h	<0.001
0h – 3h	<0.001
1h – 2h	<0.001
1h – 3h	<0.001
2h – 3h	0.112
3mM Ca^{2+} + Low TTX	
0h – 1h	<0.001
0h – 2h	<0.001
0h – 3h	<0.001
1h – 2h	0.001
1h – 3h	0.003
2h – 3h	1.00

Supplementary Table 2 Repeated-measures ANOVA
Mauchly's Test of Sphericity could not be assumed, Greenhouse-Geisser test was used for within subject effect to correct for non-sphericity of the data. All pairwise comparisons were two-sided, and were corrected using Boneferroni.
