# Supplementary material for: Purkinje cell axonal swellings enhance action potential fidelity and cerebellar function
Source: Nat Commun. 2021 Jul 5;12:4129. doi: 10.1038/s41467-021-24390-4 (PMC8257784; doi:10.1038/s41467-021-24390-4)
Supplement: Supplementary file 3 — Description of Additional Supplementary Files [file 41467_2021_24390_MOESM3_ESM.pdf]

Description of Additional Supplementary Files

File Name: Supplementary Movie 1

Description: Formation of an axon swelling along an axon in *low TTX*, imaged at 5-minute intervals.

File Name: Supplementary Movie 2

Description: Light-sheet imaging of cleared tissue displays orientation of Purkinje cells and locations of bright axonal swellings within a lobule of cerebellar vermis.
