# Supplementary material for: Purkinje cell axonal swellings enhance action potential fidelity and cerebellar function
Source: Nat Commun. 2021 Jul 5;12:4129. doi: 10.1038/s41467-021-24390-4 (PMC8257784; doi:10.1038/s41467-021-24390-4)
Supplement: Supplementary file 6 — Reporting Summary [file 41467_2021_24390_MOESM6_ESM.pdf]

nature research

Corresponding author(s): Alanna Watt

Last updated by author(s): Jun 1, 2021

Reporting Summary

Nature Research wishes to improve the reproducibility of the work that we publish. This form provides structure for consistency and transparency in reporting. For further information on Nature Research policies, see our Editorial Policies and the Editorial Policy Checklist.

Statistics

For all statistical analyses, confirm that the following items are present in the figure legend, table legend, main text, or Methods section.

n/a	Confirmed	
☐	☒	The exact sample size (*n*) for each experimental group/condition, given as a discrete number and unit of measurement
☐	☒	A statement on whether measurements were taken from distinct samples or whether the same sample was measured repeatedly
☐	☒	The statistical test(s) used AND whether they are one- or two-sided<br>*Only common tests should be described solely by name; describe more complex techniques in the Methods section.*
☐	☒	A description of all covariates tested
☐	☐	A description of any assumptions or corrections, such as tests of normality and adjustment for multiple comparisons
☐	☒	A full description of the statistical parameters including central tendency (e.g. means) or other basic estimates (e.g. regression coefficient) AND variation (e.g. standard deviation) or associated estimates of uncertainty (e.g. confidence intervals)
☐	☒	For null hypothesis testing, the test statistic (e.g. *F*, *t*, *r*) with confidence intervals, effect sizes, degrees of freedom and *P* value noted<br>*Give P values as exact values whenever suitable.*
☒	☐	For Bayesian analysis, information on the choice of priors and Markov chain Monte Carlo settings
☒	☐	For hierarchical and complex designs, identification of the appropriate level for tests and full reporting of outcomes
☐	☒	Estimates of effect sizes (e.g. Cohen's *d*, Pearson's *r*), indicating how they were calculated

Our web collection on statistics for biologists contains articles on many of the points above.

Software and code

Policy information about availability of computer code

Data collection	Electrophysiology was collected using custom-designed acquisition in Igor Pro 6.37. Imaging was acquired running ScanImage 3.7 running in Matlab 2011B. Lightsheet Z.1 with the Zen 2014 SP1 (black edition) software was used to image the whole cerebellum. Eye monitoring for VOR experiments was done with ISCAN system eye-tracking software. Custom acquisition routines in Igor Pro are available upon reasonable request from the corresponding author.
Data analysis	Electrophysiology was analyzed using custom-designed analysis routines in Igor Pro 8. Imaging was analyzed with Fiji/ImageJ2 version 2.0.0-rc-69/1.52p or Zen 2014 SP1. Lightsheet images processing was done using Zen 2.5 (blue edition). Imaris file converter and stitching 9.2.1 software was used to convert and stitch the images, and analysis was then completed using Imaris 9.3.0 software. For axonal reconstructions, we used Neurolucida 10 software. EM images were analyzed using Fiji/ImageJ2 version 2.0.0-rc-69/1.52p or Zen 2014 SP1. Eye tracking was analyzed in Matlab. Statistical analysis was performed in Microsoft SPSS 27. Monte Carlo simulation was performed in Python 3.6, and is available to download. Custom analysis routines in Igor Pro are available upon reasonable request from the corresponding author.

For manuscripts utilizing custom algorithms or software that are central to the research but not yet described in published literature, software must be made available to editors and reviewers. We strongly encourage code deposition in a community repository (e.g. GitHub). See the Nature Research guidelines for submitting code & software for further information.

Data

Policy information about availability of data

All manuscripts must include a data availability statement. This statement should provide the following information, where applicable:

- Accession codes, unique identifiers, or web links for publicly available datasets
- A list of figures that have associated raw data
- A description of any restrictions on data availability

The authors declare that the data supporting the findings of this study are available from the corresponding author upon reasonable request. Source data are provided as a Source Data file.

Field-specific reporting

Please select the one below that is the best fit for your research. If you are not sure, read the appropriate sections before making your selection.

☒ Life sciences ☐ Behavioural & social sciences ☐ Ecological, evolutionary & environmental sciences

For a reference copy of the document with all sections, see nature.com/documents/nr-reporting-summary-flat.pdf

Life sciences study design

All studies must disclose on these points even when the disclosure is negative.

Sample size	No statistical methods were used to pre-determine sample sizes but our sample sizes are similar to those reported in previous publications (e.g. Grubb et al., Nature, 2010, Ljungberg et al., Frontiers in Cellular Neuroscience, 2016). Data was collected and analyzed and if a great deal of variance across animals was observed (such as for behaviour like Rotarod, as shown by Brooks et al., Genes, Brain and Behavior, 2004), additional data was collected. Data was repeated at least 3 times for each conditions.
Data exclusions	Data was in general not excluded. However, for some analysis, such as for myelin thickness measurements, data where myelin thickness could not be assayed well was excluded. This was because of preparation artefacts leading to poor quality myelin in some of our images. For electrophysiological recordings, recordings were excluded if they were too noisy for action potentials to be determined above the noise. This was pre-determined based on temperature fluctuations of no more than 1.5 C.
Replication	Data was repeated at least 3 times for different conditions. All attempts at replication were successful.
Randomization	Data was randomized by condition. Typically 3 conditions were tested, which were randomly interspersed. Data was tested in both males and females, and differences across sex were examined.
Blinding	Since behavioral data was not collected across genotypes, there was no blinding needed for this. Since electrophysiological data for Fig. 1 and 2 were acquired with visual guidance, meaning that the experimenter could identify the axon, blinding was not typically possible. Data was analyzed blind to condition.

Reporting for specific materials, systems and methods

We require information from authors about some types of materials, experimental systems and methods used in many studies. Here, indicate whether each material, system or method listed is relevant to your study. If you are not sure if a list item applies to your research, read the appropriate section before selecting a response.

Materials & experimental systems

n/a	Involved in the study	
☐	☒	Antibodies
☒	☐	Eukaryotic cell lines
☒	☐	Palaeontology and archaeology
☐	☒	Animals and other organisms
☒	☐	Human research participants
☒	☐	Clinical data
☒	☐	Dual use research of concern

Methods

n/a	Involved in the study	
☒	☐	ChIP-seq
☒	☐	Flow cytometry
☒	☐	MRI-based neuroimaging

Antibodies

Antibodies used	Mouse anti-CASPR, 1:200, Antibodies Incorporated, Davis, CA, US, Cat#75-001<br>Rabbit anti-IP3R, 1:200, Abcam, Cambridge, UK, abID#5804<br>donkey anti-mouse Alexa 594 1:500, Life Technologies, Carlsbad, CA, USA, product # A12203<br>donkey anti-rabbit Alexa 594 1:500, Jackson Immunoresearch Labs, West Grove, PA, USA, product # 711-585-152

	mouse anti-Calbindin (1:7000 mouse monoclonal, Sigma C9848)<br>donkey anti-rabbit coupled to Alexa Fluor-488 (1:500, Life Technologies, product # A32790)<br>goat anti-mouse Cy3 (1:200; Life Technologies, product # A10521)
Validation	Antibodies were tested at a range of concentrations and optimal concentrations were chosen based on the literature and our preliminary testing.<br><br>Mouse anti-CASPR,Antibodies Incorporated, Davis, CA, US, Cat#75-001<br>Knock-out validated by UCDavid/NIH NeuroMab facility. Use: IHC.<br><br>Rabbit anti-IP3R, Abcam, Cambridge, UK, abID#5804<br>Validated in mouse cerebellar Purkinje cells, Halbach et al., Cerebellum, 2017. Use: IHC.<br><br>mouse anti-Calbindin (mouse monoclonal, Sigma C9848)<br>Validated in rodent cerebellum (Long et al., PNAS, 2014; Figuero et al., Disease models & mechanisms, 2016). Use: IHC.<br><br>All secondary antibodies were validated by repeating the immunohistochemistry in parallel while omitting the primary antibody as a negative control.

Animals and other organisms

Policy information about studies involving animals; ARRIVE guidelines recommended for reporting animal research

Laboratory animals	Male and female mice were used for all studies. Mouse were housed in 12/12 light/dark cycle, at 20-24 C, with 40-60% humidity. We used pcp2-tau-eGFP mice16,17 to characterize the functional properties of axonal swellings as well as for time-lapse visualization of their formation and for Rotarod and Erasmus Ladder behavioral studies. C57BL/6J mice were used for vestibular ocular reflex (VOR) behavioral study. Mice were used at 1-2 months of age for all behavioral studies. Acute slices were prepared from young juvenile mice (postnatal (P)9-14), when axonal swellings are numerous.
Wild animals	Study did not involve wild animals.
Field-collected samples	Study did not involve field-collected samples.
Ethics oversight	All animal procedures were approved either by the McGill Animal Care committee, in accordance with guidelines established by the Canadian Council on Animal Care, or for experiments from Fig. 6k-o, by the Institutional Animal Care and Use committee at the Erasmus Medical Centre, in accordance with the Dutch Ethical Committee for Animal experiments.

Note that full information on the approval of the study protocol must also be provided in the manuscript.
